# The signaling role of extracellular ATP in co-culture of *Shiraia* sp. S9 and *Pseudomonas fulva* SB1 for enhancing hypocrellin A production

**DOI:** 10.1186/s12934-021-01637-9

**Published:** 2021-07-23

**Authors:** Xin Ping Li, Lu Lu Zhou, Yan Hua Guo, Jian Wen Wang

**Affiliations:** College of Pharmaceutical Sciences, Soochow University, Suzhou, 215123 China

**Keywords:** *Shiraia*, *Pseudomonas fulva* SB1, Extracellular ATP, Hypocrellin, Co-culture

## Abstract

**Background:**

Adenosine 5′-triphosphate (ATP) plays both a central role as an intracellular energy source, and a crucial extracellular signaling role in diverse physiological processes of animals and plants. However, there are less reports concerning the signaling role of microbial extracellular ATP (eATP). Hypocrellins are effective anticancer photodynamic therapy (PDT) agents from bambusicolous *Shiraia* fungi. The co-culture of *Shiraia* sp. S9 and a bacterium *Pseudomonas fulva* SB1 isolated from *Shiraia* fruiting bodies was established for enhanced hypocrellin A (HA) production. The signaling roles of eATP to mediate hypocrellin biosynthesis were investigated in the co-culture.

**Results:**

The co-culture induced release of eATP at 378 nM to the medium around 4 h. The eATP release was interdependent on cytosolic Ca^2+^ concentration and reactive oxygen species (ROS) production, respectively. The eATP production could be suppressed by the Ca^2+^ chelator EGTA or abolished by the channel blocker La^3+^, ROS scavenger vitamin C and NADPH oxidase inhibitor diphenyleneiodonium chloride (DPI). The bacterium-induced H_2_O_2_ production was strongly inhibited by reactive blue (RB), a specific inhibitor of membrane purinoceptors, but dependent on the induced Ca^2+^ influx in the co-culture. On the other hand, the application of exogenous ATP (exATP) at 10–300 µM to *Shiraia* cultures also promoted fungal conidiation and HA production, both of which were blocked effectively by the purinoceptor inhibitors pyridoxalphosphate-6-azophenyl-2′, 4′-disulfonic acid (PPADS) and RB, and ATP hydrolase apyrase. Both the induced expression of HA biosynthetic genes and HA accumulation were inhibited significantly under the blocking of the eATP or Ca^2+^ signaling, and the scavenge of ROS in the co-culture.

**Conclusions:**

Our results indicate that eATP release is an early event during the intimate bacterial–fungal interaction and eATP plays a signaling role in the bacterial elicitation on fungal metabolites. Ca^2+^ and ROS are closely linked for activation of the induced ATP release and its signal transduction. This is the first report on eATP production in the fungal–bacterial co-culture and its involvement in the induced biosynthesis of fungal metabolites.

**Graphic abstract:**

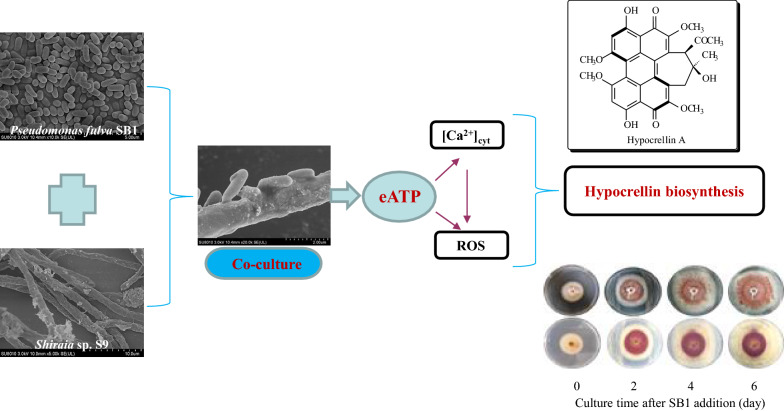

**Supplementary Information:**

The online version contains supplementary material available at 10.1186/s12934-021-01637-9.

## Background

Adenosine 5′-triphosphate (ATP) is usually recognized as a universal intracellular energy currency to support energy-requiring biochemical reactions in cells, and also function as a signaler outside the plasma membrane for several physiological processes [1]. Animal cells have the ability to produce extracellular ATP (eATP) to regulate growth, immune response, apoptosis, neurotransmission and muscle contraction [2, 3]. eATP was found to bind and activate two classes of cell surface receptors, ligand-gated ion channel P2X and G-protein-coupled P2Y receptors to generate second messengers [4]. Emerging evidence indicates eATP is involved in plant growth and development, including the regulation of membrane potential and stomatal movement, growth of root hairs and pollen tubes, gravitropism and abiotic/biotic stress responses [5]. DORN1, plant receptor for eATP, is a lectin receptor kinase, structurally different from animal ATP receptors [6]. eATP initiates the early physiological responses, such as triggering Ca^2+^ influx, stimulating generation of reactive oxygen species (ROS), and up-regulating expression of mitogen activated protein kinase (MAPK) gene, and later responses such as induced defense gene expression and disease resistance [7]. Although the role of eATP signaling in innate immunity has been well documented in both animals and plants, relatively little is known about eATP signal in microbes. The presence of eATP was observed recently in various human pathogenic bacteria *Acinetobacter*, *Aggregatibacter*, *Pseudomonas*, and *Klebsiella* [8, 9] and intestinal bacteria [10]. Ding and Tan found that eATP induced dispersal of a periodontal associated bacterium *Fusobacterium nucleatum* with enhanced virulence to elicit inflammation in periodontal disease [11]. eATP was reported as a damage-associated molecular pattern (DAMP) to induce the influx of cytosolic free calcium ([Ca^2+^]_cyt_) and activate the MAPK Tmk1 for hyphal regeneration of a filamentous fungus *Trichoderma atroviride* under mechanical damage [12, 13]. Although there was a report of exogenous ATP (exATP) to enhance tautomycetin in *Streptomyces griseochromogenes* [14], less reports have been found concerning the signaling role of eATP on the biosynthesis of microbial secondary metabolites.

Hypocrellins, the main perylenequinones of *Shiraia* fungi, are new non-porphyrin photosensitizer in photodynamic therapy (PDT) for cancers [15] and immunodeficiency virus [16]. Our previous study revealed that some eliciting strategies including light/dark shift (24: 24 h, 200 lx) and ultrasound exposure (0.28 W/cm^2^ at 40 kHz) were successful to enhance hypocrellin production of *Shiraia* [17, 18]. In our previous study [19], a bacterium *Pseudomonas fulva* SB1 from *Shiraia* fruiting bodies was found to increase hypocrellin production significantly. The established co-culture system for *Shiraia* with *P. fulva* SB1 presented a higher production of hypocrellin A (HA) 325.87 mg/L, about 3.20-fold of that in axenic culture [20]. Furthermore, we found the expression of ATP-binding cassette (*ABC*) of *Shiraia* sp. S9 was up-regulated, about 3.1-fold of the mono-culture control. More evidence supported that ABC is the one of carriers for the active transport of ATP from intracellular stores into the extracellular matrix [21, 22]. On the other hand, the signaling of ROS and Ca^2+^/calmodulin (CaM) have been validated during the application of Triton X‑100 and fungal elicitor on *Shiraia* for hypocrellin production [23, 24]. Since the increased levels of [Ca^2+^]_cyt_ and ROS have been proved the early signaling steps of eATP in both animal and plant cells [7], we hereby hypothesized that eATP may be involved in the induced responses during the bacterial–fungal interaction. Recently, bacterial–fungal co-culture which mimics the natural microbe communities is becoming an effective strategy to expand microbial chemodiversity or improve metabolite production. The microbial metabolites (259 compounds) induced in co-cultures are subdivided by Arora et al. into 9 significant clusters in three groups of “non-nitrogenated compounds”, “non-alkaloidic nitrogenated compounds” and “alkaloidic compounds” [25]. The induction by co-culture was revealed to be the result of the microbial biotransformation [26], activation of cryptic biosynthetic pathways [27] or the increased histone acetylation [28]. However, little information is available regarding chemical signals for the compound induction by co-culture. As a follow-up to our efforts to elucidate the signaling events on eliciting hypocrellin biosynthesis [18, 20, 24], we examined the changes of eATP in the co-culture of *Shiraia* sp. S9 with the bacterium *P. fulva* SB1. We also explored the relationship between eATP and induced ROS or Ca^2+^ influx, and their mediation on hypocrellin biosynthesis. Understanding the signaling role of eATP in the bacterium–host interaction may provide new insight in the induction mechanisms and lead to the development of novel co-culture strategy for fungal metabolite production.

## Results

### eATP release in the co-culture

**Fig. 1:**
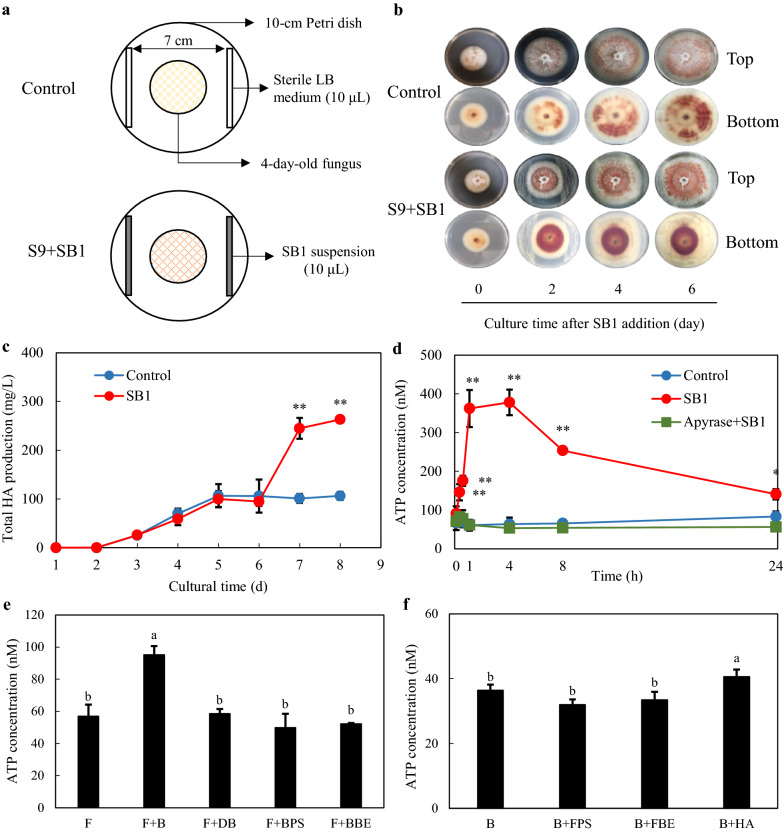
Hypocrellin A (HA) production and ATP release in the co-culture of *Shiraia* sp. S9 and *P. fulva* SB1. **a** Scheme of the *in vitro* dual culture plate confrontation assay. **b** The effects of live SB1 on the growth and red pigments secretion of S9 in PDA plate. The 4-day-old fungal colony was treated with SB1 for 2–6 days. The bacterial suspension (10 µL) was streaked in two parallel straight lines, approximately 7 cm apart from each other. The culture was maintained on PDA at 28 °C. **c** The total HA production of S9 in the co-culture. The co-culture was maintained in 150-mL flask containing 50 mL of the liquid medium at 150 rpm and 28 °C for 8 d. **d** ATP release in the co-culture. The 6-day-old fungal mycelia were treated with SB1 (400 cells/mL) and incubated at 150 rpm and 28 °C. Apyrase at 2 U/mL was added to the culture at 1 h prior to the addition of SB1. **e** The released ATP in the fungal culture with live SB1 (B, 400 cells/mL), heat-killed SB1 (DB, 400 cells/mL), the crude bacterial polysaccharide (BPS) and bacterial broth extracts (BBE) at 100 mg/mL for 1 h. **f** The released ATP in bacterial culture with fungal polysaccharides (FPS) and the ethyl acetate extracts (FBE) at 100 mg/mL and HA (5 mg/mL) treatment for 1 h. Values are mean ± SD from three independent experiments (*p < 0.05 and **p < 0.01 vs. control). Different letters above the bars mean significant differences (*p* < 0.05)

The fungal-bacterial confrontation assay (Fig. 1a) was used to investigate the effect of the bacterium *P. fulva* SB1 on the hypocrellin accumulation of host fungus *Shiraia* sp. S9 in PDA medium. After 2–6 days of co-culture, the secretion of red pigments of mycelia of S9 was observed (Fig. 1b). The total HA production in shake flask culture was measured to 263.25 mg/L, a 2.48-fold of mono-culture of S9 on day 8 (Fig. 1c). A significant increase of the eATP was observed after 15 min and its concentration peaked at 378 nM around 4 h (Fig. 1d). To determine the source of eATP production, we measured eATP release in the fungal culture (F) treated with live bacterium (B), the bacterial broth extracts (BBE), crude bacterial polysaccharide (BPS) as well as heat-killed bacterium (DB) (Fig. 1e). The eATP concentration was increased only by live bacterium (F + B in Fig. 1e), whereas the treatment of bacterial extracts (BBE and BPS) did not alter the eATP concentration. Conversely, we measured the eATP production in bacterial cultures treated by fungal polysaccharides (FPS) or fungal broth extracts (FBE) as well as HA at 5 mg/mL (Fig. 1f). The eATP concentration was only improved slightly by HA. These results suggested that the release of eATP depended on the presence of live bacterium and fungus in the co-culture.

### eATP-induced fungal HA production and conidiation

**Fig. 2:**
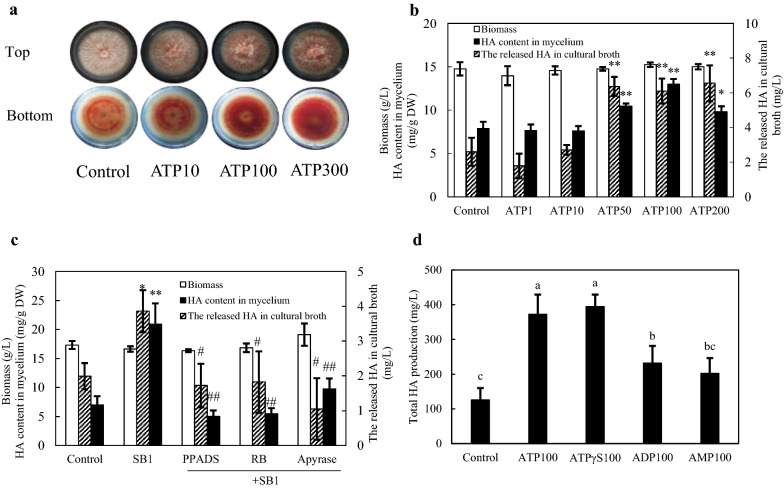
The effect of ATP on hypocrellin A (HA) production of *Shiraia* sp. S9. **a** The effects of exogenous ATP (exATP) on the growth and red pigments secretion of *Shiraia* sp. S9 in PDA plate. S9 was treated with exATP and incubated at 28 °C for 10 days. **b** The effects of exATP on HA production in submerged culture. exATP was added on day 6 during the 8-day-culture of S9. **c** The effect of purinoceptor inhibitors and apyrase on SB1-induced HA production. The 6-day-old fungal mycelia were incubated with inhibitors pyridoxalphosphate-6-azophenyl-2′, 4′-disulfonic acid (PPADS) and reactive blue (RB) at 10 µM and apyrase (2 U/mL) for 1 h before SB1 addition and cultured for additional 2 days. **d** The effects of ATP analog (ATPγS) and derivatives (ADP and AMP) on HA production in submerged culture. exATP, ATPγS, ADP and AMP were added respectively at 100 µM on day 6 during the 8-day-culture of S9. Values are mean ± SD from three independent experiments (*p < 0.05 and **p < 0.01 vs. control, ^#^*p* < 0.05 and ^##^*p* < 0.01 vs. SB1 treatment). Different letters above the bars mean significant differences (*p* < 0.05)

exATP at 10–300 µM was applied to the solid culture of S9 and the secretion of red pigments was increased (Fig. 2a). In a liquid culture, both the intracellular and extracellular HA was enhanced by exATP (50–200 µM) and dose-dependent (Fig. 2b). To verify the effect of ATP, both the purinoceptor inhibitor pyridoxalphosphate-6-azophenyl-2′, 4′-disulfonic acid (PPADS) or reactive blue (RB) at 10 µM, and an ATP hydrolyzer apyrase at 2 U/mL were used to block eATP response in the co-culture. As shown in Fig. 2c, the intracellular and extracellular HA were decreased significantly by apyrase or the purinoceptor inhibitors in the co-culture. On the other hand, we conducted an additional control experiment with the non-hydrolyzable form of ATP (ATPγS) and ATP derivatives (ADP and AMP) to induce HA production (Fig. 2d). ATPγS increased HA production to a similar level compared with that induced by exATP while ADP or AMP induced much lower levels of HA increase, indicating that ATP hydrolysis is not required for the elicitation on HA production.

**Fig. 3:**
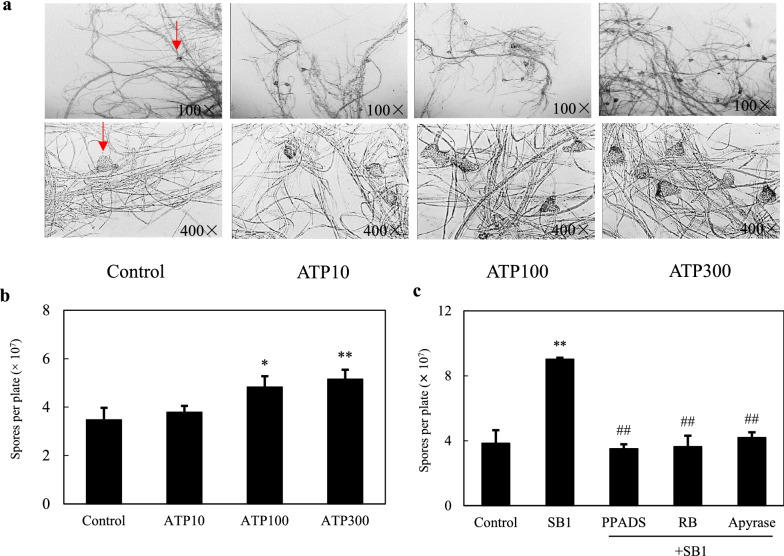
The effect of ATP on aerial mycelial growth **a** and conidiation **b** of *Shiraia* sp. S9. in PDA plate. Arrow (red) indicates pycnidium. **c** The effect of purinoceptor inhibitors and apyrase on SB1-induced fungal conidiation. S9 was treated with pyridoxalphosphate-6-azophenyl-2′, 4′-disulfonic acid (PPADS) and reactive blue (RB) at 10 µM or apyrase (2 U/mL) for 30 min, then placed in the center of 10-cm PDA plate. The culture was maintained on PDA at 28 °C for 8 d. Values are mean ± SD from three independent experiments (*p < 0.05 and **p < 0.01 vs. control, ^#^*p* < 0.05 and ^##^*p* < 0.01 vs. SB1 treatment)

Simultaneously, we found that the number of pycnidium and the conidia concentration of S9 was increased dose-dependently after exATP treatment at 100, 300 µM, respectively (Fig. 3a, b). In the fungal-bacterial confrontation assay, live bacterium SB1 increased the fungal conidiation by 134.1 % (SB1 in Fig. 3c). Both the treatment of the purinoceptor inhibitors and ATP hydrolyzer apyrase significant suppressed the induced conidiation (Fig. 3c), indicating that the SB1-induced conidiation was modulated by eATP signal.

### Effect of eATP on SB1-induced ROS generation

**Fig. 4:**
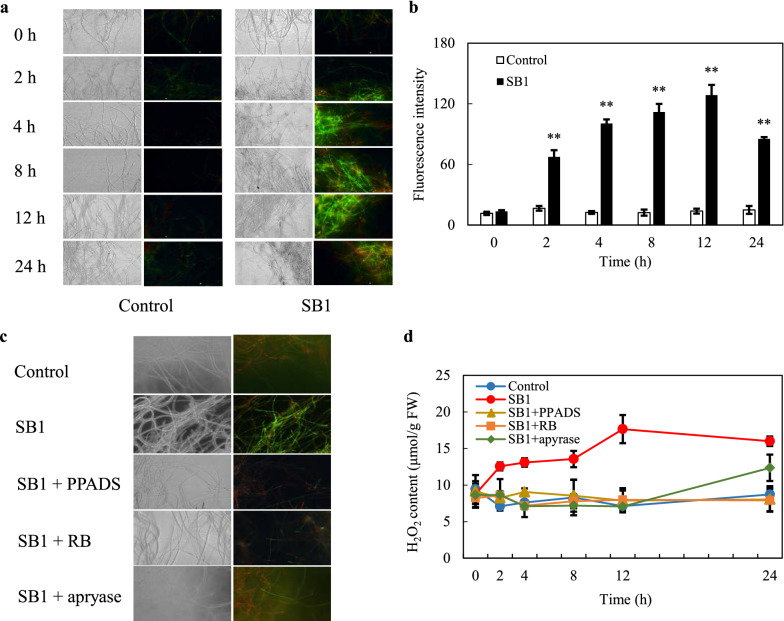
Effect of eATP on SB1-induced ROS accumulation of *Shiraia* sp. S9. **a** Bright-field images (left) and fluorescence microscopy (right) of 2, 7-dichlorodihydrofluorescein diacetate (DCFH-DA)-stained mycelia (×400). **b** Reactive oxygen species (ROS) accumulation in S9 after the addition of SB1 at 400 cells/mL. **c** The effect of pyridoxalphosphate-6-azophenyl-2′, 4′-disulfonic acid (PPADS), reactive blue (RB) and apyrase on SB1-induced ROS accumulation and **d** H_2_O_2_ content of S9. The 6-day-old fungal mycelia were incubated with 10 µM DCFH-DA for 30 min. The SB1 treatment was the same as specified in Fig. 1. The dosages of PPADS, RB and apyrase were the same as specified in Fig. 2. Values are mean ± SD from three independent experiments (**p < 0.01 vs. control)

As shown in Fig. 4a, the green fluorescence detected by 2, 7-dichlorodihydrofluorescein diacetate (DCFH-DA) in fungal mycelia was found after SB1 addition within 2 h. The fluorescence intensity reached to a maximum level (9.32-fold of control) around 12 h (Fig. 4b). The SB1-induced green fluorescence was almost completely suppressed by PPADS (10 µM), RB (10 µM) or apyrase (2 U/mL) (Fig. 4c). The H_2_O_2_ content in SB1 treated mycelia reached to a peak level of 17.65 µmol/g fresh weight (FW) at 12 h, which was 2.48-fold of the control level (Fig. 4d). But H_2_O_2_ contents in fungal mycelia were decreased by 55.3 %, 54.7% and 59.9 % by PPADS, RB and apyrase at 12 h, respectively (Fig. 4d). These results suggested that the induction of ROS generation by SB1 was dependent on eATP release in the co-culture.

### Effect of eATP on SB1-induced Ca^2+^ influx

**Fig. 5:**
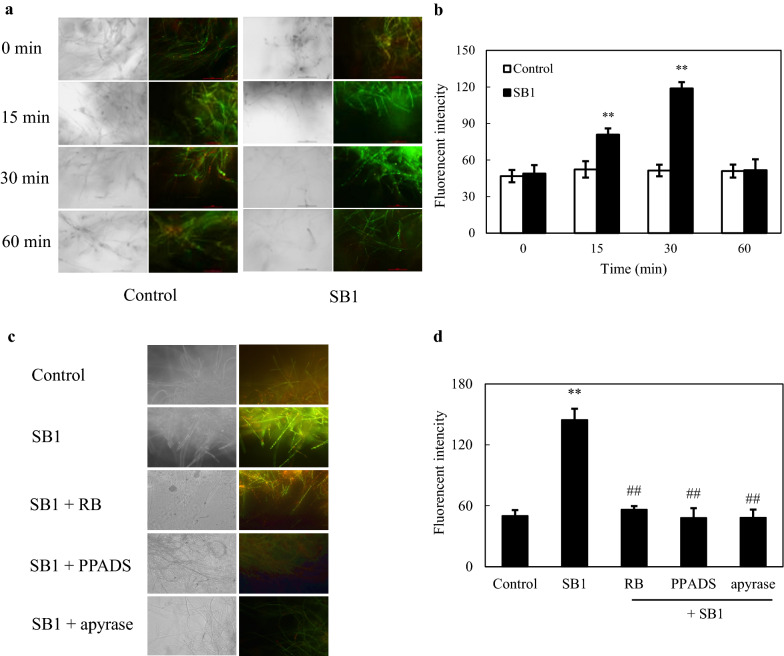
Effect of eATP on SB1-induced Ca^2+^ influx of *Shiraia* sp. S9. **a** Bright-field images (left) and fluorescence microscopy (right) of Fluo-3 AM-stained mycelia (400 ×). **b** Ca^2+^ accumulation in S9 after the addition of SB1 at 400 cells/mL. **c** Bright-field images (left) and fluorescence microscopy (right) and **d** fluorescent intensity of Fluo-3 AM-stained mycelia (400 ×) treated by pyridoxalphosphate-6-azophenyl-2′, 4′-disulfonic acid (PPADS), reactive blue (RB) and apyrase in the co-culture. SB1 treatment was the same as specified in Fig. 1. The dosages of PPADS, RB and apyrase were the same as specified in Fig. 2. Values are mean ± SD from three independent experiments (**p < 0.01 vs. control, ^##^*p* < 0.01 vs. SB1 treatment)

The Ca^2+^ accumulation in SB1 treated fungal mycelia was directly observed by using the fluorescent cell-permeant calcium indicator Fluo-3 AM. The greener fluorescence of fungal mycelia was observed after the SB1 addition (Fig. 5). The enhanced fluorescent signal was detected within 15 min and rose to a peak (2.31-fold of control) around 30 min, and then dropped back to the initial control level after 30 min (Fig. 5a, b). The green fluorescence was blocked by PPADS, RB and apyrase (Fig. 5c) and the induced-elevation of cytosolic free calcium was decreased by 66.7%, 61.0% and 66.5%, separately after SB1 addition for 30 min (Fig. 5d). These results indicated that SB1-induced Ca^2+^ influx was dependent on eATP in the co-culture.

### Dependence of eATP, ROS and Ca^2+^

**Fig. 6:**
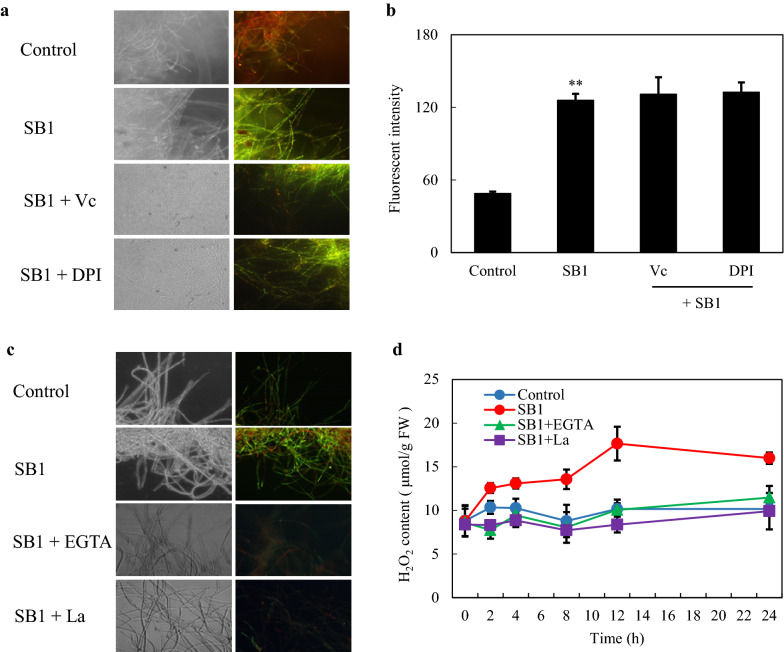
**a** Bright-field images (left) and fluorescence microscopy (right) of Fluo-3 AM-stained mycelia (×400) treated by vitamin C (Vc) or diphenyleneiodonium chloride (DPI) in the co-culture. **b** Ca^2+^ accumulation in S9 treated by Vc or DPI in the co-culture. Vc (0.1 mM) or DPI (5 µM) was added to the culture at 1 h prior to the addition of SB1 (400 cells/mL). **c** Bright-field images (left) and fluorescence microscopy (right) of 2, 7-dichlorodihydrofluorescein diacetate (DCFH-DA)-stained mycelia treated by EGTA (5 mM) and La^3+^ (×400). **d** H_2_O_2_ content of S9 treated by EGTA or La^3+^ in the co-culture. EGTA (5 mM) and La^3+^ (2 mM) were added to the culture at 1 h prior to the addition of SB1 (400 cells/mL). Values are mean ± SD from three independent experiments (**p < 0.01 vs. control)

**Fig. 7:**
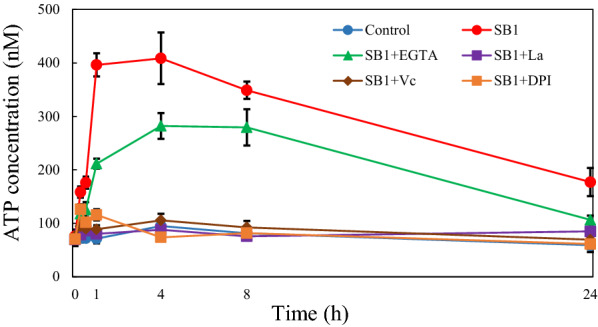
The SB1-induced ATP release and its dependence on Ca^2+^ and ROS in *Shiraia* sp. S9. The SB1 treatment was the same as specified in Fig. 1. The dosages of vitamin C (Vc), diphenyleneiodonium chloride (DPI), EGTA and La^3+^ were the same as specified in Fig. 6. Values are mean ± SD from three independent experiments

To further investigate on the interaction among SB1-induced ROS and Ca^2+^, vitamin C (Vc, 0.1 mM) and NADPH oxidase inhibitor diphenyleneiodonium chloride (DPI, 5 µM) were used to inhibit ROS generation, while Ca^2+^ chelator (EGTA at 5 mM) and membrane channel blocker (La^3+^ at 2 mM) were used to inhibit the Ca^2+^ influx (Fig. 6). When SB1-induced ROS was eliminated by Vc and DPI, there were not any notable changes of Ca^2+^ in fluorescent signal and its intensity (SB1 + Vc or + DPI vs. SB1 in Fig. 6a, b). However, the ROS production in mycelia were reduced after Ca^2+^ signaling was blocked by EGTA or La^3+^ (SB1 + EGTA or + La^3+^ vs. SB1 in Fig. 6c, d). The results suggested that the ROS production may occur downstream of Ca^2+^ influx in the co-culture. Furthermore, the eATP production in the co-culture was effectively suppressed to control level after ROS inhibition by Vc and DPI, or blocking Ca^2+^ influx by La^3+^ (Fig. 7). While pre-treatment with EGTA before SB1 addition only partially decreased the eATP release. These results indicated that Ca^2+^ influx and ROS generation may also contribute to the regulation of eATP efflux in the co-culture.

### Effect of eATP on expressions of biosynthetic genes for HA

**Fig. 8:**
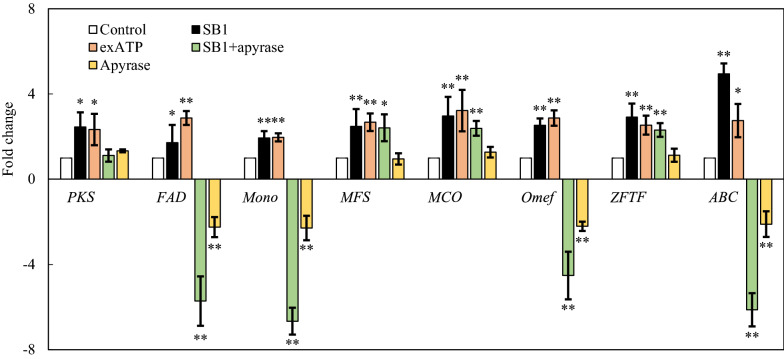
Effect of eATP on the expressions of hypocrellin A (HA) biosynthetic genes of *Shiraia* sp. S9. The co-culture method was the same as specified in Fig. 1. Exogenous ATP (exATP) and apyrase treatment and their concentration were the same as specified in Fig. 2. The culture was maintained in 150-mL flask containing 50 mL of the liquid medium at 150 rpm and 28 °C for 8 days. Values are mean ± SD from three independent experiments (*p < 0.05 and **p < 0.01 vs. control)

To investigate on the mechanism of eATP on SB1-induced HA production, the expressions of eight genes for HA biosynthesis were analyzed by qRT-PCR after 24 h of co-culture (Fig. 8). The genes encoding polyketide synthase (*PKS*), FAD/FMN-dependent oxidoreductase (*FAD*), monooxygenase (*Mono*), multicopper oxidase (*MCO*), *O*-methyltransferase (*Omef*), zinc finger transcription factor (*ZFTF*), major facilitator superfamily (*MFS*) and ATP-binding cassette transporter (*ABC*) were all up-regulated, about 2.45-, 1.71-, 1.94-, 2.97-, 2.53-, 2.91-, 2.47- and 4.94-fold of control, respectively. Simultaneously, exATP at 100 µM also increased their expressions with the similar trend of live SB1. However, after eATP release in co-culture was hydrolyzed by apyrase, the expression of *PKS* was dropped back to control level, whereas *FAD*, *Mono*, *Omef* and *ABC* expressions were down-regulated (4.52-6.66-fold of control).

**Fig. 9:**
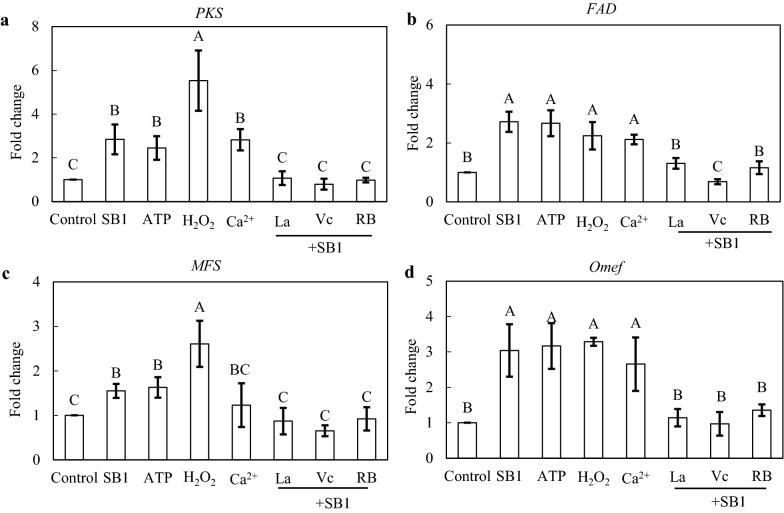
The effect of SB1, exogenous ATP (exATP), Ca^2+^ and H_2_O_2_ on the expressions of hypocrellin A (HA) biosynthetic genes. The SB1 and ATP treatments were the same as specified in Fig. 2. H_2_O_2_ at 1 mM and Ca^2+^ (CaCl_2_) at 2 g/L were added on day 6 during the 7-day-old culture of S9. The dosages of La^3+^, vitamin C (Vc) and reactive blue (RB) were the same as specified as mentioned in Figs. 5 and 6. The culture was maintained in 150-mL flask containing 50 mL of the liquid medium at 150 rpm and 28 °C for 7 days. Values are mean ± SD from three independent experiments. Different letters above the bars mean significant differences (*p* < 0.05)

**Fig. 10:**
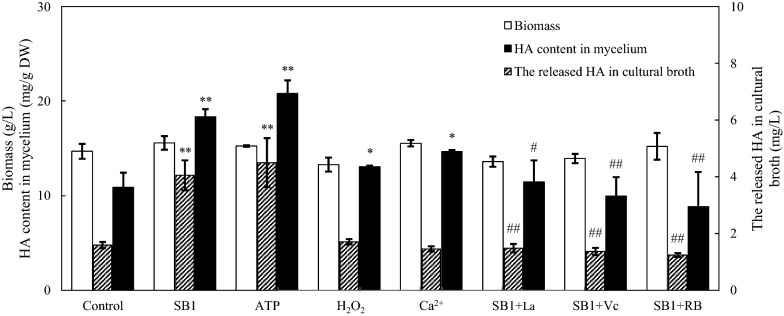
Effect of SB1, ATP, Ca^2+^ and H_2_O_2_ on hypocrellin A (HA) production of *Shiraia* sp. S9. The SB1 and ATP treatments were the same as specified in Fig. 7. H_2_O_2_ at 1 mM and Ca^2+^ (CaCl_2_) at 2 g/L were added on day 6. The dosages of La^3+^, vitamin C (Vc) and reactive blue (RB) were the same as specified in Figs. 5 and 6. The culture was maintained in 150-mL flask containing 50 mL of the liquid medium at 150 rpm and 28 °C for 8 days. Values are mean ± SD from three independent experiments (*p < 0.05 and **p < 0.01 vs. control, ^#^*p* < 0.05 and ^##^*p* < 0.01 vs. SB1 treatment)

To further investigate the influence of eATP, ROS and Ca^2+^ influx on HA biosynthesis in co-culture, the exogenous donors (exATP, H_2_O_2_ and Ca^2+^) or specific inhibitors (RB, Vc and La^3+^) were used (Fig. 9). After the addition of exATP at 100 µM, H_2_O_2_ at 1 mM or Ca^2+^ at 2 g/L for 24 h, significant increase in the transcript level of *PKS* (145.2-453.1 %), *FAD* (111.6-166.9 %), *MFS* (23.0-160.8 %) and *Omef* (165.6-228.9 %) were observed respectively. On the other hand, the enhanced expressions of these genes induced by live SB1 were inhibited after Ca^2+^, ROS, eATP signaling was blocked by La^3+^, Vc or RB, respectively (SB1 + La^3+^, + Vc or + RB vs. SB1 in Fig. 9). Simultaneously, HA contents in mycelium were all increased by 68.9 %, 91.4%, 20.1% and 34.9% after SB1, exATP, H_2_O_2_ and Ca^2+^ treatment, respectively (Fig. 10). The released HA in cultural broth were increased by 154.7% and 183.0% by SB1 and exATP, respectively. However, both the intracellular and extracellular HA productions were significantly decreased by La^3+^, Vc or RB in the co-culture (SB1 + La^3+^, + Vc or + RB vs. SB1 in Fig. 10). Taken together, these results suggested strongly that the SB1-induced HA biosynthesis was mediated by eATP, ROS and Ca^2+^ signaling.

## Discussion

eATP has been recognized as an important signaling molecule that affects a broad range of biological processes in multicellular organisms like plants and animals [1]. Recent studies show that bacteria, yeasts and fungi can also release ATP into the extracellular compartment or the culture medium. An earlier report by Ivanova et al. (2006) showed some Gram-negative α-proteobacteria and γ-proteobacteria could release eATP from micromolar to millimolar concentrations [29]. Some *Enterococcus* strains from mice and human feces, urine, and skin could secrete eATP at 1–3 µM in the cultural supernatant, but two bacterial strains (*Escherichia coli* and *Staphylococcus aureus*) failed to show eATP secretion [30]. eATP production was found to be dependent on bacterial glycolysis, respiration and growth phase [8, 31]. Although eATP released by periodontal associated bacteria or commensal bacteria in the intestines has been reported to be involved in the periodontal or intestinal inflammation [11, 32], the purpose and significance of bacterial eATP is still poorly understood. Fungi such as *Candida albicans* and yeast *Saccharomyces cerevisiae* were found to secret ATP in the presence of glucose [33] or against the treatment of antifungal salivary histatins [34]. eATP from *S. cerevisiae* was elevated during growth and acted as the intercellular signal to induce and synchronize yeast sporulation [35]. Medina-Castellanos et al. (2014; 2018) demonstrated a filamentous fungus *T. atroviride* could regulate wound responses, conidiation and hyphal regeneration by eATP [12, 13]. In this study, we found that eATP was released in co-culture of a bambusicolous fungus *Shiraia* sp. S9 and a fruiting body‑associated bacterium *P. fulva* SB1 to 146–378 nM during 4 h, and fully suppressed by adding apyrase at 2 U/mL for the hydrolysis of ATP (Fig. 1d). These results suggested the production of eATP was triggered by the co-culture. This is the first report on eATP production in the fungal–bacterial co-culture.

In our study, the treatment of BBE, BPS and DB exhibited no capacity to induce eATP production in the fungal culture (Fig. 1e). The effect of live bacteria and cell-free bacterial broth on the fungus *Arthrinium* c.f. *saccharicola* was investigated [36]. The fungus growth was inhibited by live bacterium *Pseudoalteromonas piscida* whereas the cell-free culture broth of the bacterium induced the changes of fungal metabolite profiles and improved the antibacterial activity of the fungus. The signaling molecules produced by the bacterium may contribute to the enhanced fungal bioactivity. Furthermore, Schroeckh et al. (2009) demonstrated the intimate physical contact of *Aspergillus nidulans* and *S. hygroscopicus* in the co-culture was required for induction of silent polyketide metabolism in *Aspergillus* [27]. Those results support that both diffusible signaling molecules from the bacterium and intimate bacterial contact can have physiological roles during the co-cultures. In the present study, eATP production in the bacterial culture was also not elicited by FBE and FPS (Fig. 1f), indicating that the live bacterium and fungus were essential for the secretion of eATP to the medium. In our previous study on the co-culture of *Shiraia* sp. S9 and *P. fulva* SB1 [20], both the direct physical contact and fragmentation of hyphae of *Shiraia* sp. S9 were observed. In plants, eATP acts as DAMP signal during herbivory, mechanical damage or pathogen attack [5]. The increased eATP levels were also found in damaged hyphae of *T. atroviride* by using a scalpel [12]. Thus, further studies are needed to verify whether eATP is DAMP signal associated with intimate bacterial–fungal interactions in the co-culture.

In the present study, fungal conidiation was activated after the co-cultivation with the bacterium SB1 (Fig. 3c). The concentration of conidia was increased from 3.47 × 10^7^ to 5.16 × 10^7^ spores/plate after exATP treatment (300 µM) (Fig. 3b). The SB1-induced fungal conidiation in co-culture was completely blocked by nucleotide receptor antagonists (PPADS and RB at 10 µM) and ATP hydrolase (apyrase at 2 U/mL) (Fig. 3c). These results were consistent with the induced conidiation found in *T. atroviride* in response to mechanical damage by a scalpel [12, 13]. Our results demonstrated that exATP (10–300 µM) promoted significantly the secretion of HA in PDA plate (Fig. 2a) and the HA production in shake flasks (Fig. 2b). Furthermore, the suppression of live bacterium-induced HA production by nucleotide receptor antagonist RB or ATP hydrolase apyrase was observed in Fig. 2c. Therefore, taken together, our results suggested the existence of eATP receptors and the regulatory role of eATP on fungal conidiation and HA biosynthesis in the co-culture.

Our study demonstrated the effective blockage of fungal ROS production by RB, PPADS and apyrase (Fig. 4c, d). The results suggest that eATP is a key signal for ROS mediating responses to the live bacterium. Based on the time courses of eATP release (Fig. 1d) and H_2_O_2_ production (Fig. 4d), the peak value of eATP level induced by the bacterium SB1 was detected earlier (4 h) than H_2_O_2_ production (12 h). However, the full suppression of eATP in the co-culture by DPI (NADPH oxidase inhibitor) and VC (ROS scavenger) suggested that ROS generation also affected eATP levels (Fig. 7). In our previous studies, ROS generation has been induced to mediate the enhancement of HA production by abiotic elicitors including ultrasound stimulation [18], Triton X-100 treatment [24] and light/dark exposure [17]. Although our present results are still not sufficient to determine whether eATP acts as a signal upstream or downstream of ROS in the co-culture, eATP and ROS are necessary signal molecules involved in metabolite production in *Shiraia* under the elicitation.

**Fig. 11:**
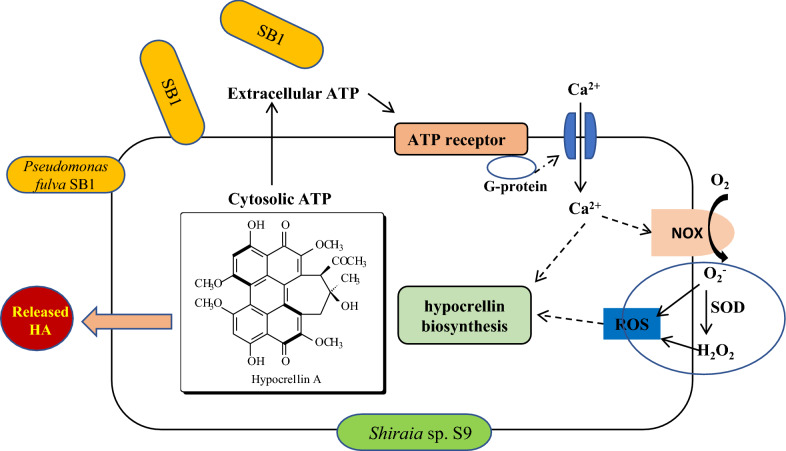
A hypothetical extracellular ATP (eATP) signal pathway leading to hypocrellin A (HA) biosynthesis in co-culture of *Shiraia* sp. S9 and *Pseudomonas fulva* SB1 (ROS, reactive oxygen species; NOX, NADPH oxidase)

Plant and animal cells have the ability to detect eATP through activation of receptors to trigger a transient increase in intracellular Ca^2+^ as key “danger” signals in wounding [5] and the inflammatory processes [3]. The activation of P2X and P2Y purinergic receptors by ATP triggered a transient increase in intracellular Ca^2+^ by promoting extracellular Ca^2+^ influx or endoplasmatic reticulum Ca^2+^ release in neural differentiation [37]. In *Arabidopsis*, DORN1, a lectin receptor kinase was bound to eATP with high affinity and required for the induced intracellular Ca^2+^ [6]. Our data showed the accumulation of intracellular Ca^2+^ (Fig. 5) was observed after SB1 treatment for 30 min. The application of nucleotide receptor antagonists (PPADS or RB) suppressed fully the SB1-activated Ca^2+^ accumulation (Fig. 5d), indicating the regulation role of eATP in the Ca^2+^ accumulation. In turn, the released eATP was completely blocked by Ca^2+^ membrane channel blocker La^3+^ and significantly decreased by Ca^2+^ chelator (Fig. 7). Although the strong calcium dependence for eATP production was revealed, current evidence is still insufficient to make a clear conclusion that eATP secretion is downstream of Ca^2+^ influx in the co-culture or vice versa. Furthermore, Song et al. (2006) reported that the eATP induced the accumulation of superoxide was dependent on cytosolic Ca^2+^ concentration in *Arabidopsis* [38]. ROS also mediated the cytosolic Ca^2+^ elevation by activating plasma membrane hyperpolarization-activated Ca^2+^ influx channels [39]. In present study, we found that the inhibition of ROS production by Vc or DPI did not affect Ca^2+^ accumulation (Fig. 6a), but chelation or block of Ca^2+^ remarkably decreased ROS production in co-culture (Fig. 6c, d). These results suggested that ROS generation might occur downstream of the Ca^2+^ influx in the co-culture (Fig. 11). In *Shiraia* sp. Slf14, the transcriptional levels of Ca^2+^ sensor-encoding genes (*cam*, *can*, and *crz*1) and biosynthetic genes (*pks*, *omef* and *hydroxylase*) for HA was upregulated by Ca^2+^ induction [23]. In this study, the expressions of HA biosynthetical genes (*PKS*, *FAD*, and *Mono*) were upregulated markedly in co-culture or by exATP at 100 µM (Fig. 8). Both the expression of HA biosynthetic genes and the HA accumulation in the co-culture were significantly inhibited under the blocking of the eATP or Ca^2+^ signaling by RB or La^3+^, and the scavenge of ROS by Vc (Figs. 9 and 10). These results indicate that eATP can act as a signal message in the Ca^2+^-mediated signal transduction leading to the biosynthesis of HA in the co-culture (Fig. 11).

## Conclusions

We demonstrated that transient ATP release was an early event in the response of fungal host *Shiraia* sp. S9 to a fruiting body-associated bacterium *P. fulva* SB1. The eATP signal was sensitive to RB and PPADS and may act as DAMP signal associated with intimate bacterial–fungal interactions. eATP was closely linked to Ca^2+^ influx and ROS generation to become an essential early event in the activation of fungal conidiation and the elicitation of HA production. These small molecular signals could contribute to the bacterial–fungal communication leading to the transcript induction of HA biosynthetic genes. To the best of our knowledge, this study is the first to demonstrate the elevation of eATP by the interaction of bacteria and fungi. These findings increase our understanding of signal role of eATP in microbes and intimate bacterial-fungal interaction for developing co-culture strategy in the production of desired fungal metabolites.

## Materials and methods

### Strains, media, and culture conditions

*Shiraia* sp. S9 and the bacterium *P. fulva* SB1 were isolated from the fresh *Shiraia* fruiting bodies in our previous work [20] and deposited in China General Microbiological Culture Collection Center (registered as CGMCC16369 and CGMCC 13931 respectively). SB1 was stored in Luria-Bertani (LB) slant (pH7.2) at 4 °C and initially grown on LB medium in a Petri dish at 37 °C for 2 days. S9 was kept on a potato dextrose agar (PDA) slant at 4 °C and initially grown on PDA medium in a Petri dish at 28 °C for 10 days. As a preculture, 50-mL of modified liquid medium (100 g/L potato, 20 g/L starch, 4 g/L NaNO_3_, 1.5 g/L KH_2_PO_4_, 0.5 g/L CaCO_3_ and 0.01 g/L VB_1_, pH 6.3) inoculated with 10^7^ spores/mL was used. The preculture was maintained at 28 °C for 2 days with shaking at 150 rpm and then transferred (1 mL) into a 150-mL flask containing 50 mL of the same liquid medium at 150 rpm and 28 °C for 8 days.

### Co-culture of *Shiraia* sp. S9 and *P. fulva* SB1

Fungal-bacterial co-cultivation on PDA plates were performed as previously described [20]. In details, the S9 strain was initially grown on PDA for 8 days at 28 °C. A small piece of S9 (5 mm × 5 mm) was placed in the center of a PDA plate (10 cm) and incubated at 28 °C for 4 days in the dark. Then, 10 µL of the SB1 suspension (co-cultivation) or fresh sterile LB broth (control) was streaked in two parallel straight lines, about 7 cm apart from each other (Fig. 1a). To establish the co-cultivation in *Shiraia* mycelium culture, live SB1 at 400 cells/mL was added to 6-day-old culture and maintained at 150 rpm and 28 °C [20].

### Preparation of elicitors and inhibitor solutions

To determine the source of eATP in the co-culture, we used the extracts of the bacterium SB1 or the fungal strain S9 as elicitors to induce eATP release from the culture. Bacterial cells (SB1) and cultural broth were harvested after 12 h by centrifugation at 12,000 rpm for 10 min. The supernatant was extracted by threefold volume of ethyl acetate and re-dissolved in ethanol as bacterial broth extracts (BBE). The crude SB1 polysaccharide (BPS) was prepared according to our previously described method [20]. For preparation of the fungal extracts, the cultural broth was collected from 6-day-old mycelium culture by filtration with 400-mesh filter membrane (Dongkang, Tianjin, China) and extracted by threefold volume of 95 % ethanol. The precipitate was collected and dialyzed to remove small molecules (< 3,500 Da), and finally lyophilized to obtain the fungal polysaccharides (FPS). The ethyl acetate extracts of the cultural broth were evaporated in vacuo to afford a residue (FBE).

To determine the changes of eATP in the culture of S9 after the bacterial elicitation, the live SB1 (B, 400 cells/mL), autoclaved SB1 suspension (DB, 400 cells/mL), BPS (100 mg/L) and BBE (100 mg/L) were added to 6-day-old culture of fungal S9, respectively. To determine the changes of eATP in the bacterial culture, FPS (100 mg/L), FBE (100 mg/L) and standard HA (5 mg/mL) were added to live SB1 (400 cells/mL) in 50 mL shake flasks filled with 10 mL modified liquid medium. eATP concentration was detected after 1 h of the treatment.

To determine the dependence of bacteria SB1 elicitation on eATP, some specific antagonists were applied to the cultures, i.e. RB (Yuanye Biotech., Shanghai, China) at 10 µM as an inhibitor of eATP signal transduction across the plasma membrane, PPADS (Abcam, Cambridge, MA, USA) at 10 µM as the purinoceptor inhibitor, and apyrase (Sigma-Aldrich, St Louis, MO, USA) at 2 U/mL as an enzyme for hydrolyzing ATP. These inhibitors and their dosages were chosen based on previous studies [40, 41]. The inhibitors were added to the culture at 1 h prior to the addition of SB1. Additional control experiments were conducted in the mycelium culture with a nonhydrolyzable form of ATP, ATPγS (Sigma‐Aldrich, St Louis, MO, USA) at 100 µM, and two hydrolyzed ATP derivatives, ADP and AMP (Yuanye Biotech., Shanghai, China) at 100 µM to determine the effects of ATP hydrolysis on the elicitation.

### Detection of extracellular ATP

The eATP concentration was determined by a fluorometric method using the luciferin-luciferase ATP assay kit (Beyotime Biotech., Haimen, Jiangsu, China) as reported by Wu et al. [41]. The fermentation broth (500 µL) was collected from each of the shake flasks at selected time intervals for the luminance measure by a GloMax 20/20 luminometer (Promega, USA).

### Detection of ROS generation

ROS generation in hyphae of *Shiraia* sp. S9 was detected by using DCFH-DA (Beyotime Biotech., Haimen, Jiangsu, China) under the fluorescence microscopy (BX51, Olympus, Tokyo, Japan) with excitation wavelength at 480 nm and emission wavelength at 520 nm [42]. The content of hydrogen peroxide (H_2_O_2_) was measured as described previously [43]. To analysis the signaling role of ROS in the co-culture, 0.1 mM Vc or 5 µM DPI was added 1 h prior to the addition of SB1 in the culture.

### Measurement of intracellular Ca^2+^

The change of intracellular Ca^2+^ concentration in fungal pellets was detected by the Ca^2+^-sensitive probe Fluo-3-AM (Beyotime Biotech., Haimen, Jiangsu, China). The fungal pellets were incubated at 4 °C for 2 h in PBS containing 0.2 mM CaCl_2_ and 5 µM Fluo-3-AM [40]. Subsequently, the pellets were incubated in the fungal medium for another 2 h, and then subjected to various treatments. After the treatments, the pellets were collected and photographed by fluorescence microscopy (BX51, Olympus, Tokyo, Japan) with excitation wavelength at 480 nm and emission wavelength at 515 nm. The integration of fluorescence intensity was defined as the relative intracellular Ca^2+^ concentration level [40]. Ca^2+^ chelator EGTA at 5 mM and membrane channel blocker La^3+^ (with LaCl_3_) at 2 mM were applied to determine the signaling role of Ca^2+^ in the co-culture [41]. The inhibitors were added 1 h prior to the addition of SB1 in the culture.

### Quantification of conidia and HA production

The conidia were collected in sterile water and quantified by hemocytometer. The intracellular and extracellular HA were extracted based on our previous report [24]. HPLC analysis was carried out in a reverse-phase Agilent 1260 HPLC system (Agilent Co., Wilmington, DE, USA) with Agilent HC-C18 column (250 mm × 4.6 mm). HA was quantified with genuine standards (Chinese National Compound Library, Shanghai, China).

### Quantitative real-time polymerase chain reaction (qRT-PCR) analysis

The primer sequences of HA biosynthesis related genes and 18S ribosomal RNA as internal reference gene were listed in Additional file 1: Table S1. The qRT-PCR was performed according to the method described in our previous study [44]. The transcriptional expression levels of genes were calculated from cycle threshold values by using the 2^−△△CT^ method described by Zhang et al. [45].

### Statistical analysis

Student’s *t*-test was used as a significance test to compared the means between two groups. One-way analysis of variance (ANOVA) was used to assess the significance of differences of the means among multiple groups. All results are expressed as Mean ± Standard Deviation (SD). The level of significance was set at *p* < 0.05.

## Supplementary Information

**Additional file 1: Table S1.** Primers and relevant information of reference and target genes. F: forward primer, R: reverse primer.

## Data Availability

All data generated or analyzed during this study are included in this published article and its Additional files.
