# Supplementary material for: The signaling role of extracellular ATP in co-culture of Shiraia sp. S9 and Pseudomonas fulva SB1 for enhancing hypocrellin A production
Source: Microb Cell Fact. 2021 Jul 23;20:144. doi: 10.1186/s12934-021-01637-9 (PMC8305905; doi:10.1186/s12934-021-01637-9)
Supplement: Supplementary file 1 — Additional file 1: Table S1. Primers and relevant information of reference and target genes. F: forward primer, R: reverse primer. [file 12934_2021_1637_MOESM1_ESM.pdf]

Additional file 1

The signaling role of extracellular ATP in co-culture of *Shiraia* sp. S9 and *Pseudomonas fulva* SB1 for enhancing hypocrellin A production

Xin Ping Li, Lu Lu Zhou, Yan Hua Guo, Jian Wen Wang

✉ Jian Wen Wang

jwwang@suda.edu.cn; bcjwwang@gmail.com

Table S1. Primers and relevant information of reference and target genes. F: forward primer, R: reverse primer.

Genes symbol	Gene name	Sequence
18S	Reference gene	F: GAAAGTTAGGGGATCGAAGA<br>R: TAGTCGGCATAGTTTACGGT
PKS	Polyketide synthase	F: TGCTGAGGTAGCAGTCAAGC<br>R: TTATGCTACGGTCGTCGCTC
FAD	FAD/FMN-containing dehydrogenase	F: TGTGACCGCCATCACCTTAC<br>R: TTGTCGTATGGGTGGGAAGC
Mono	Salicylate 1-monooxygenase	F: TCTCGGGGAATTATGGCACG<br>R: ACAACCGTTCTCGCATCAGT
MFS	Major facilitator superfamily	F: TCCCGTAGCCTTGCTTTCTG<br>R: CCGGCTTCTTCTTGACGCTA
MCO	Multicopper oxidase	F: TATGGCGCTACGAGTGGAC<br>R: ACTCCCTGGCCGATAACGTA
Omef	*O*-methyltransferase	F: GAACTACCTGAAGGCACGCT<br>R: GCTCGGAAGGATACTCGCTC
ZFTF	Zinc finger transcription factor	F: GAACACCGTCGCAAGATTCG<br>R: TCATTGGCATCGCTTGGAGT
ABC	ATP-binding cassette	F: GACTTGAGCCTATCCGCCTC<br>R: AGAGTCGCCTCTGTGATCCT
